# Isolation of xylose-utilizing yeasts from oil palm waste for xylitol and ethanol production

**DOI:** 10.1186/s40643-023-00691-y

**Published:** 2023-10-13

**Authors:** N. Kusumawati, S. H. Sumarlan, E. Zubaidah, A. K. Wardani

**Affiliations:** 1 Department of Agroindustrial Technology, Faculty of Agricultural Technology, Universitas Brawijaya, Jl. Veteran, Malang, 65145 Indonesia; 2 Department of Food Technology, Faculty of Agricultural Technology, Widya Mandala Catholic University Surabaya, Jl. Dinoyo 42-44, Surabaya, 60625 Indonesia; 3 Department of Agricultural Engineering, Faculty of Agricultural Technology, Universitas Brawijaya, Jl. Veteran, Malang, 65145 Indonesia; 4 Department of Food Science and Biotechnology, Faculty of Agricultural Technology, Universitas Brawijaya, Jl. Veteran, Malang, 65145 Indonesia

**Keywords:** Yeast, Xylose, Xylitol, Ethanol, Lignocellulosic-biomass

## Abstract

The energy crisis triggers the use of energy sources that are renewable, such as biomass made from lignocellulosic materials, to produce various chemical compounds for food ingredients and biofuel. The efficient conversion of lignocellulosic biomass into products with added value involves the activity of microorganisms, such as yeasts. For the conversion, microorganisms must be able to use various sugars in lignocellulosic biomass, including pentose sugars, especially xylose. This study aims to isolate xylose-utilizing yeasts and analyze their fermentation activity to produce xylitol and ethanol, as well as their ability to grow in liquid hydrolysate produced from pretreated lignocellulosic biomass. Nineteen yeast isolates could grow on solid and liquid media using solely xylose as a carbon source. All isolates can grow in a xylose medium with incubation at 30 °C, 37 °C, 42 °C, and 45 °C. Six isolates, namely SLI (1), SL3, SL6, SL7, R5, and OPT4B, were chosen based on their considerable growth and high xylose consumption rate in a medium with 50 g/L xylose with incubation at 30 °C for 48 h. Four isolates tested, namely SLI (1), SL6, SL7, and R5, can produce xylitol in media containing xylose carbon sources. The concentration of xylitol produced was determined using high-pressure liquid chromatography (HPLC), and the results ranged from 5.0 to 6.0 g/L. Five isolates tested, namely SLI (1), SL6, SL3, R5, and OPT4B, can produce ethanol. The ethanol content produced was determined using gas chromatography (GC), with concentrations ranging from 0.85 to 1.34 g/L. Three isolates, namely SL1(1), R5, and SL6, were able to produce xylitol and ethanol from xylose as carbon sources and were also able to grow on liquid hydrolyzate from pretreated oil palm trunk waste with the subcritical water method. The three isolates were further analyzed using the 18S rDNA sequence to identify the species and confirm their phylogenetic position. Identification based on DNA sequence analysis revealed that isolates SL1(1) and R5 were *Pichia kudriavzevii*, while isolate SL6 was *Candida xylopsoci*. The yeast strains isolated from this study could potentially be used for the bioconversion process of lignocellulosic biomass waste to produce value-added derivative products.

**Graphical Abstract:**

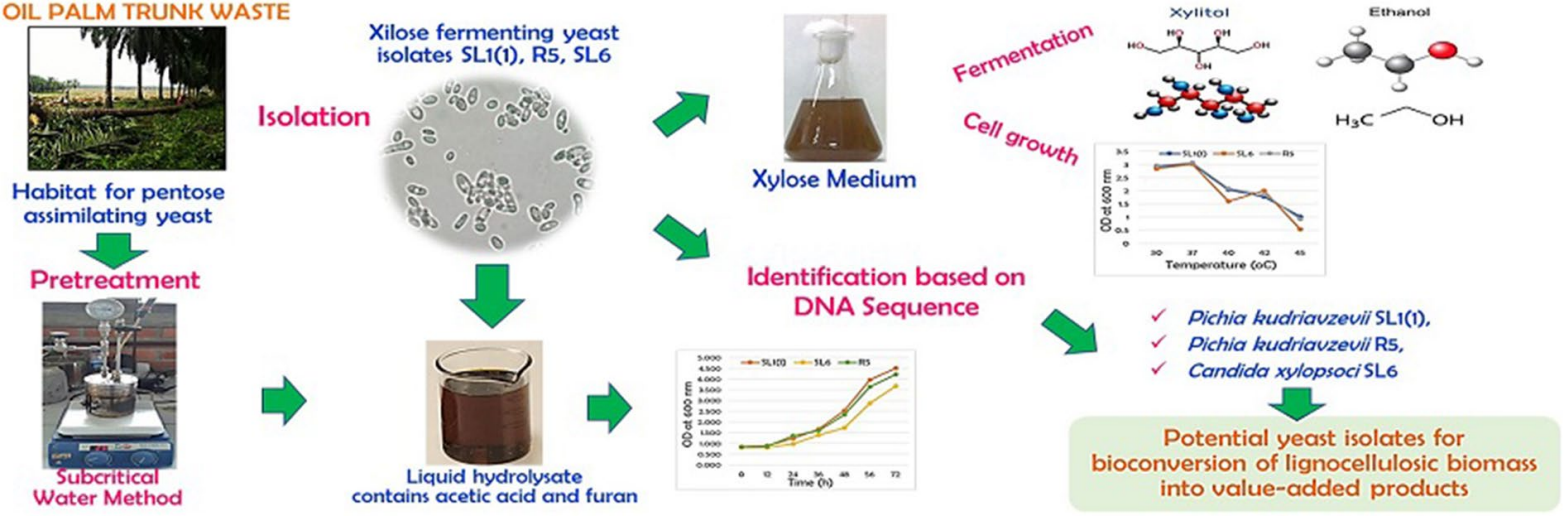

## Introduction

The energy crisis prompted research and development targeted at producing chemicals and fuel from renewable lignocellulosic feedstocks obtained from agricultural and forestry wastes sustainably. As a renewable resource, lignocellulosic biomass obtained from the forest or crop residue is a significant source of biotechnologically created organic compounds and fuel for the future. The components of lignocellulosic biomass include cellulose, hemicellulose, and lignin. Cellulose is a homopolymer of glucose, whereas hemicellulose comprises hexose and pentose sugars. The quantities of distinct sugars vary depending on the raw material (Kim et al. 2010; Tye et al. 2016). Hemicellulose hydrolysate is usually made up of sugars from pentose (xylose and arabinose) and some hexose sugars (glucose, mannose, and galactose) (Singh et al. 2011). Xylose represents the most widespread pentose in the biosphere and the second-largest sugar after glucose. Xylose is the most plentiful sugar in hemicelluloses and is predominantly plant sugar.

Yeast and bacterial species are the most commonly used agents for conducting xylose bioconversion to biofuels and high-value chemicals. Unfortunately, bacteria have various disadvantages, such as bacteriophage lysis sensitivity, limited volume of cells, which raises the expense of biomass separateness, poor thermotolerance, and difficulties with bacterial biomass utilization. As a result, yeast is being studied more than bacteria for xylose bioconversion. Because of the practical significance of xylose bioconversion to high-value compounds and the general curiosity in the metabolism of the second most abundant carbon molecule in the biosphere, the principal metabolic pathway(s) employed in yeasts for xylose use are of significant interest. One of the requirements for optimal use of lignocellulosic biomass is complete substrate consumption. All forms of sugar in hemicellulose and cellulose must be utilized as substrates to produce value-added products such as xylitol or biofuels. The use of hemicellulose derivative sugars, which has been extensively researched, is a substrate for producing xylitol and ethanol.

Xylitol (C_5_H_12_O_5_) is a polyol that has a high sweetness level, with only 40% of calories from sucrose, a low glycemic index, does not trigger cancer (non-carcinogenicity), inhibits caries (cariostatic), and has antidiabetic potential (Amo et al. 2011; Kishore et al. 2012). Conventionally, xylitol production is carried out by hydrogenating pure d-xylose solutions using catalysts at high temperatures and pressures. Because of the operating conditions and the need for the purity of the xylose used, traditional processes are expensive. Currently, xylitol has a strong demand in the global market—more than 120,000 tons per year with a relatively high value—spurred research to search for low-cost production processes. One alternative is to use the biotechnology process with raw materials in the form of waste that contain xylose sugar, which can be converted into xylitol, such as lignocellulosic biomass. The intricate metabolic control of xylitol formation includes xylose transport, the manufacture of essential enzymes, and cofactor replenishment. Therefore, screening naturally occurring xylose-utilizing microbes is a practical and successful method to find xylitol-producing organisms with commercial applications.

Plant biomass is expected to supply nearly one-quarter of the world's energy by 2035, making biofuels a renewable alternative to fossil fuels (Kalyani et al. 2017). First-generation bioethanol is made from agricultural products, including corn and sugarcane. The most significant disadvantage of the initial ethanol from biomass is that it uses crops for food to make fuel, which may result in food crises and higher food prices. Second-generation ethanol from biomass is generated using lignocellulosic material, which includes resources from forests, agricultural waste, and municipal trash. These natural resources are plentiful, inexpensive, and do not compete with food supplies (Kalyani et al. 2017; Nguyen et al. 2017; Guerrero et al. 2018).

In addition to utilizing various types of sugar and having fermentation activity to produce value-added products, the requirements for strains to be used in lignocellulosic biomass bioconversion are their tolerance to inhibitory compounds produced during the pretreatment of the biomass. Several lignin and sugar breakdown products are generated throughout the pretreatment process, which may adversely influence enzyme hydrolysis and fermentation by microbial cells, including yeast (Jönsson et al. 2013).

Throughout this investigation, yeast isolates capable of metabolizing xylose were obtained from oil palm waste degraded and dispersed throughout the plantation. Oil palm waste includes a lot of cellulose and hemicellulose, which may be hydrolyzed to generate pentose and hexose sugars. Many xylanase- and cellulase-producing strains are discovered in habitats rich in lignocellulosic biomass waste, such as oil palm waste. Previous studies have isolated many molds with cellulolytic and xylanolytic activity from oil palm biomass residue (Kusumawati et al. 2020). These molds can degrade cellulose and xylan in palm oil waste to produce pentose and hexose sugars, which can then be utilized by microbes, including yeasts, as substrates to generate value-added products. This research aimed to isolate yeast that can metabolize xylose and test their fermentation activity to produce xylitol and ethanol and their ability to grow in liquid hydrolysate produced from pretreated lignocellulosic biomass.

## Materials and methods

### Materials

Degraded and scattered oil palm trunk waste in oil palm estate was used as a source of yeast isolate obtained from oil palm plantations in Lampung and Riau, Indonesia. Samples from oil palm plantations were taken and put in plastic bags, then sent from the plantation to the laboratory location at room temperature (28–31 °C) for 24 h (from Lampung) and 48 h (from Riau). The lignocellulosic biomass used as a source of hydrolysate was obtained from chopped oil palm trunks from a 27-year-old plant.

Peptone, agar for microbiology, NaCl, D( +)-xylose, D( +)-glucose, were obtained from Merck KGa, Darmstadt, Germany. Xylitol, xylose, glucose standard and H_2_SO_4_, HCl, acetonitrile (HPLC grade), was obtained from Sigma-Aldrich, USA.

### Sample preparation and pretreatment

Biomass from degraded and scattered oil palm trunk was crushed with a chopper to obtain particles with a diameter of about 0.5 mm and then stored in a sterile container at room temperature until used as a sample for the enrichment and yeast isolation steps. The OPT was washed with running tap water and dried under sunlight for about 2 days. OPT were then milled, screened through a 20-mesh-sized aluminum sieve, and dried in an oven at 105 °C for 24 h. The dry biomass particles were then stored in a freezer until used in pretreatment experiments to produce hydrolyzate. The method for pretreatment of oil palm trunk waste was subcritical water. The experiment was carried out at 170 °C, 400 psi pressure for 20 min, and the ratio of dry sample to water was 7:1 (ml/g). The hydrolysate obtained was separated into liquid and solid phases. The resulting liquid phase contained xylose and glucose at 4.859 and 4.900 g/L, respectively. The liquid hydrolysate also contains furfural and acetic acid at 0.293 and 2.224 g/L, respectively (data not shown). The hydrolysate is then concentrated using a vacuum evaporator at 50 °C at a pressure of 90–100 mBar so that the volume becomes 20% of the initial volume. The concentrated solution was used for further experiments.

### Enrichment and isolation of xylose-utilizing yeasts

One g of the above samples was inoculated in 3% xylose broth enrichment medium (0.3 g/L xylose; 0.025 g/L peptone with initial pH was 6.5) and incubated at 30 °C for 48 h in a shaking water bath at 100 rpm. The enriched samples were serially diluted using sterile saline (NaCl 0.85%). A sample of 0.1 mL of each dilution was distributed over 3% xylose agar medium, and the cultivated plates were then incubated at 30 °C for 48 h (Navnit and Unati 2015). All yeast colonies have been selected and examined under a microscope. The cultures were re-streaked until single colonies were produced and then grown on an agar medium containing 3% xylose as a carbon source.

### Xylose assimilation tests

Xylose assimilation assays were carried out on complex media with xylose as the only carbon supply (YPX medium) (Guo et al. 2006). YPX medium contains 10 g/L yeast extract, 20 g/L peptone, 50 g/L xylose, and 15 g/L agar for solid medium or without agar for liquid medium. The pH of the medium was initially adjusted to 5.0 using 6 mol/L HCl. This test was carried out sequentially on solid and liquid media. Yeast cells were incubated for 24 h in a shaking water bath at 100 rpm at 30 °C in a medium of YPD broth (glucose, 20 g/L). The precultures' serial dilutions were performed and subsequently placed on the YPX (xylose, 20 g/L) agar plate. The total number of colonies on the plate was counted after 72 h of incubation at 30 °C. For growth in a liquid medium, a 500 µL aliquot of preculture of each strain was inoculated into Erlenmeyer containing 50 ml YPX (50 g/L xylose) and incubated in the shaking water bath at 100 rpm at 30 °C. Cell growth was monitored by measuring optical density (OD) at 600 nm after incubation for 24, 48, and 72 h, then harvested by centrifugation at 3461 × g for 30 min. Substrate consumption and product formation were analyzed by HPLC. Each isolate was tested in triplicate separately. Cell dry biomass was determined with the following procedure: 10.0 mL of liquid culture of yeast isolates was collected by centrifugation at 3461 × g for 30 min and washed twice by the sterile aquadest to produce suspensions, which were placed on pre-weighted glass bottles and dried at 105 °C to obtain constant weight.

### Growth of yeast isolates at various temperatures and in liquid hydrolysate from pretreated oil palm trunk waste

Analysis to determine the ability of yeast isolates to grow at various temperatures was performed according to the procedure by Talukder et al. (2019). Fifty µl (100-fold dilution) yeast cells from an actively developing culture in YPD broth were injected into test tubes containing 5 ml of YPX (xylose 50 g/L) broth and maintained for 72 h at different temperatures (30, 37, 40, 42, and 45 °C). After incubation for 72 h, the optical density of suspended cells was measured using a spectrophotometer (Shimadzu, Japan) at 600 nm wavelength with the YPX liquid medium as a blank.

Yeast strains capable of producing xylitol and ethanol using xylose substrates were tested for their ability to grow in liquid hydrolysate extracted from pretreated oil palm trunk waste with the subcritical water method. Fifty µl of yeast cells from actively growing cultures in YPD broth were inoculated into a test tube containing 5 ml of liquid hydrolysate from pretreated oil palm trunk waste and incubated at 30 °C and 100 rpm. The growth of yeast strains was monitored after incubation for 0, 12, 24, 36, 48, 56, and 72 h by measuring OD at a wavelength of 600 nm, with the non-inoculated liquid hydrolysate as a blank.

### Analysis of xylose and xylitol production

To find out the xylitol produced by yeast in media containing xylose as a carbon source, a test was carried out as was done by Guo et al. (2006). Yeast cells were grown in YPX (xylose 50 g/L) media for 72 h at 30 °C and 100 rpm. Liquid cultures of isolates were then spun at 6000 rpm for 30 min, and the resulting liquids were used to determine the contents of xylose and xylitol. HPLC with an Aminex® HPX-87 H Ion Exclusion Column (300 × 7.8 mm) and RI@8X detector was used to quantify the concentrations of the substrates and products. The H_2_SO_4_ with a concentration of 0.008 N was used as the mobile phase. The flow rate was set to 1.0 ml/min, and the separation temperature was 35 °C.

### Analysis of ethanol concentration

The ethanol concentration was measured using a gas chromatography according to that carried out by Martins et al. (2018). Yeast cells were grown in both YPD (glucose 100 g/L) and YPX (xylose 50 g/L) media for 72 h at 30 °C and 100 rpm. A gas chromatograph (Shimadzu GC-2010 Plus) with a Rtx-wax column (length 30 m, diameter 0.25 mm) and a flame ionization detector, one split/splitless injector, was used to determine the ethanol concentration. Nitrogen at 30 mL/min was used as a carrier gas. The injector and detector were set to 225 °C and 250 °C, respectively.

### rDNA sequence analysis

The 18S rDNA sequence analysis was performed to identify the phylogeny of the yeast (Wu et al. 2018). ITS-4: TCCTCCGCTTATTGATATGC and ITS-5: GGAAGTAAAAGTCGTAACAAGG primers were used to amplify the 18S rDNA gene. Sequence similarity searches were performed using the blast network service of the NCBI database at http://www.ncbi.nlm.nih.gov/ BLAST to identify the genus of isolated strain. Nucleotide sequence alignments were carried out using the clustal w sequence analysis program. Analysis of DNA sequences and construction of a phylogeny tree was performed using distance (neighbor-joining) methods in MEGA (ver. 6.06).

## Results and discussion

### Xylose-utilizing yeasts

**Fig. 1:**
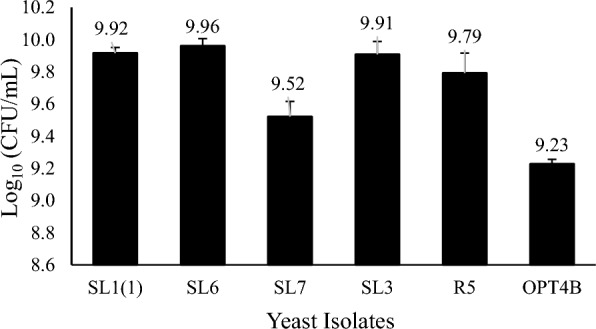
Viable cells profiles of yeast isolates. Cells were precultured overnight in liquid YPD (glucose, 20 g/L) medium in shaking water bath at 100 rpm for 30 °C and then after serial dilution was laid on the YPX (xylose, 20 g/L) agar plate. Data points were obtained from three separately replicate experiments. Differences between replicates were < 10%

**Fig. 2:**
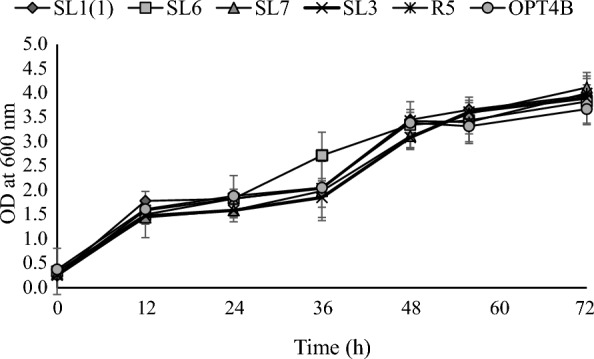
Growth profiles of yeast isolates. Cells were grown in YPX (xylose 50 g/L) media for 72 h at 30 °C and 100 rpm. Three independently replicated experiments generated data points. The variance between repetitions was < 10%

**Fig. 3:**
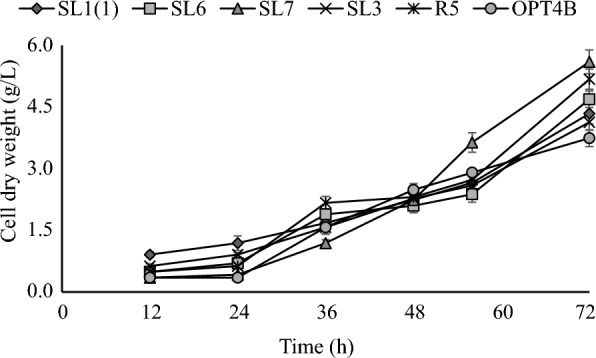
Cell dry weight profiles of yeast isolates. Cells were grown in YPX (xylose 50 g/L) media for 72 h at 30 °C and 100 rpm. Three independently replicated experiments generated data points. The variance between repetitions was < 10%

Pieces of degraded oil palm waste scattered in oil palm plantations are used as a source of yeast isolates. The isolation was initiated by enrichment in liquid media containing xylose as a carbon source and then grown in solid media with xylose carbon sources. The colonies obtained were observed macro and microscopically. Selected colonies were then purified. The pure cultures were used in further tests. Six isolates were able to assimilate xylose, as evidenced by strong growth on solid and fluid media using xylose as a single supply of carbon. The growth profile of yeast isolates was investigated by measuring the number of living cells (Fig. 1), the turbidity of growth media detected by OD at a wavelength of 600 nm (Fig. 2), and the dry weight of cells (Fig. 3). Six yeast isolates tested in this study were able to grow in media using xylose as a carbon source. Colony growth of the six isolates on agar plates with a xylose carbon source was above 9 log cfu/mL. The growth of isolates in liquid medium containing xylose with incubation at 30 °C for 72 h was recorded as OD at 600 nm ranging from 3,672 to 4.115. The biomass of the six isolates grown in the liquid medium ranged from 3.75 to 5.60 g/L.

**Fig. 4:**
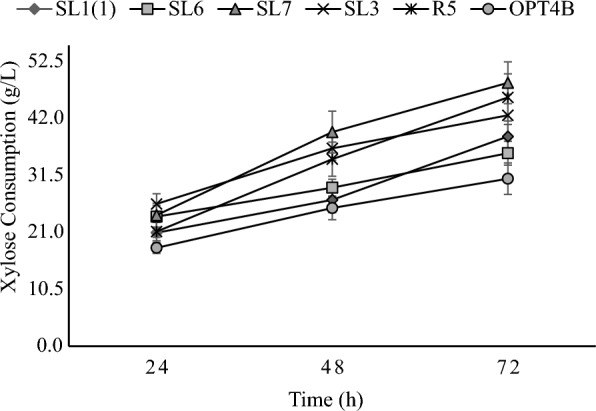
Xylose consumption profiles by yeast isolates. Cells were grown in YPX (xylose 50 g/L) media for 72 h at 30 °C and 100 rpm. Two independently replicated experiments generated data points. The variance between repetitions was < 10%

Yeast isolates grow by consuming xylose as the only carbon source in the media. The profile of xylose consumption by yeast isolated in this study is shown in Fig. 4. Data in Fig. 4 show that under aerobic conditions for 72 h of incubation at 30 °C, yeast consumes D-xylose in the range of 30.9–48.6 g/L or around 61.82–97.2% of the initial xylose in the media, which is 50 g/L. Consumption of xylose by yeast isolated in this study was lower than consumption by strains of *Candida tropicalis. Metschnikowia koreensis, Rhodotorula mucilaginosa,* and *Pichia guilliermondii* species isolated by Martins et al. (2018), which is about 90–100% of the initial xylose, i.e., 30 g/L during 72–120 h incubation at 30 °C. The strain of the species *Candida pseudorhagii, Candida shehatae, Hamamotoa lignophila,* and *Sugiyamaella sp.* isolated by Ali et al. (2017) consumed 81.3–100% of the 50 g/L xylose contained in the initial medium after 48–72 h of incubation. However, the consumption of xylose by yeast isolates obtained from this study was greater than that of strains of the species *Aureobasidium pullulans*, *Candida blankii, Issatchenkia terricola*, and *Hanseniaspora sp*. (Ali et al. 2017; Martins et al. 2018). Consumption of xylose by several yeast strains of the same species can vary greatly. For example, the *Candida tropicalis* FP strain consumed 30 g/L (100%) of xylose, while the *Candida tropicalis* S3 strain only ten g/L (33.3%) in the same medium and incubation time (Martins et al. 2018).

Numerous yeasts that can break down xylose have been identified thus far. Many ascomycete yeasts, even those that cannot digest this sugar, appear to have xylose digestion genes. Nearly all ascomycete yeasts include candidate genes for xylose reductase (XYL1), xylitol dehydrogenase (XYL2), and xylulokinase (XYL3), typically in numerous paralogs. The inability to develop on xylose while possessing the necessary genes may thus be caused by other elements, such as the control of gene expression, the specificity of an enzyme, or the inability to transport xylose (Ruchala and Sibirny 2021). Xylose assimilation in yeasts takes the sugar via an enzymatic reservoir before entering the phosphopentose route (Bettiga et al. 2008). To absorb pentose sugars, D-xylose reductase (E.C. 1.1.1.21) converts xylose to xylitol, which is then oxidized by xylitol dehydrogenase (E.C. 1.1.1.9), yielding D-xylose-5-phosphate. The Embden–Meyerhof pathway includes ribulose phosphate-3-epimerase (5.1.3.1), transaldolase (E.C. 2.2.1.2), and transketolase (E.C. 2.2.1.1) in succession transforming D-xylose-5-phosphate into glyceraldehyde-3-phosphate and fructose-6-phosphate via non-oxidative restructuring to form ethanol. NADPH must be replenished via the processes of metabolism.

Natural xylose-fermenting yeast strains such as *Candida shehatae, Kluyveromyces marxianus, Pachysolen tannophilus,* and *Pichia stipitis* have been the most thoroughly studied and are the most efficient xylose fermenters (Antunes et al. 2013, Laluce et al. 2012). Several other yeast species that are also capable of metabolizing xylose include *Aerobasidium pullulans, Candida tropicalis, Candida oleophila, Hanseniaspora guilliermondii, Issatchenkia terricola, Metschnikowia koreensis, Pichia guilliermondii,* and *Rhodotorula mucilagi*nosa (Araújo et al. 2018, Martins et al. 2018; Ruchala and Sibirny 2021). They are most recognized for being able to convert xylose to ethanol. On the other hand, some xylose-digesting yeast species have been reported to be remarkable for transforming xylose to other compounds other than ethanol. These studies also show that from the same species, the ability to metabolize xylose is not the same in different strains (Martins et al. 2018; Pilap et al. 2022). This finding highlights the necessity of studying each strain individually because they respond differently to environmental influences, even though they are from the same species.

### Growth profile of yeast isolates at different temperature

**Fig. 5:**
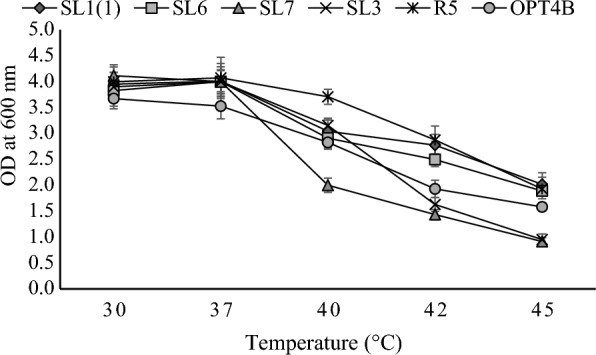
Growth profiles of yeast isolates at different temperatures. Cells were grown in YPX (xylose 50 g/L) media at 100 rpm at certain temperatures, i.e., 30, 37, 40, 42, 45 °C. Three independently replicated experiments generated data points. The variance between repetitions was < 10%

Six yeast isolates tested in this study could grow on media using xylose as a carbon supply at various temperatures from 30 to 45 °C. Although the cell density of all isolates decreased at temperatures above 37 °C, the growth of all isolates was quite significant at 42 °C so that they could be grouped as thermotolerant. The most rapid growth of isolates SL7 and OPT4B occurred at 30 °C, while isolates SL1(1), SL6, SL3 and R5 at 37 °C. The growth of all isolates at temperatures of 30 and 37 °C did not differ much, so in this study the ability of isolates to produce xylitol and ethanol was tested at the same temperature, 30 °C. The growth profile of yeast isolates at various temperatures is shown in Fig. 5.

The data in Fig. 5 show that the growth rates of all isolates at 30 and 37 °C were almost the same, but as the temperature increased from 37 to 45 °C, the cell density decreased gradually. The sharpest decrease in growth occurred in isolate SL7 from OD 3.99 at 37 °C to 2.00 at 40 °C. An increase in temperature to a certain level will make it easier for many proteins to be involved in various metabolic processes, thereby increasing the growth rate. Increasing growth temperature also affects protein movement and membrane integrity (Choudhary et al. 2016). At an increase in temperature above the tolerance of the cell, it causes the protein enzymes and cell walls to denature so that the enzymes are inactive, and membrane leakage can occur, which causes cell death.

Several researchers have isolated thermotolerant yeast strains that play a role in the bioconversion of lignocellulosic biomass into value-added products (Tanimura et al. 2012; Murata et al. 2015; Ndubuisi et al. 2018; Pongcharoen et al. 2018; Phong et al. 2019; Ulya et al. 2018; Rahman et al. 2021; Pilap et al. 2022). *Pichia kudriavzevii* is one of the most widely isolated thermotolerant yeast species in these studies, which can ferment sugar into ethanol (Ndubuisi et al. 2018; Pongcharoen et al. 2018; Ulya et al. 2018; Rahman et al. 2021; Pilap et al. 2022). Apart from *Pichia kudriavzevii*, several thermotolerant yeast strains with the ability to ferment alcohol from other species that have been studied include *Candida shehatae* (Tanimura et al. 2012), *Kluyveromyces marxianus* (Murata et al. 2015), *Meyerozyma caribbica, Saccharomyces cerevisiae, Candida tropicalis, Torulaspora globosa*, and *Pichia manshurica* (Phong et al. 2019), *Saccharomycodes ludwigii*, and *Pichia manshurica* (Pilap et al. 2022). Most of these studies tested the ability of yeast to grow at high temperatures in media with a carbon source of glucose or in hydrolyzate media extracted from lignocellulosic biomass containing various types of hexose and pentose sugars. Several thermotolerant strains of the *Candida shehatae* species studied by Tanimura et al. (2012) and *Pichia kudriavzevii* studied by Rahman et al. (2021) and Pilap et al. (2022) were found to be able to grow in media containing xylose substrates.

Using thermotolerant yeast in manufacturing industrial alcohol has numerous possible benefits: it has a high fermentation rate and production output due to its rapid metabolic activity. The ability to dissolve oxygen and other gases in the fermentation solution reduces as the temperature rises. This effect encourages the creation and continual upkeep of anaerobic circumstances essential for optimum yeast growth. As the temperature increases, the viscosity of the solution for fermentation decreases. As a result, the energy required to keep the growth media agitated is lowered. Microbe activity related to metabolism and the physical effects of stirring generate a significant quantity of heat. As a result, both the extra energy required to retain the containers at the proper temperature and the cooling needs after sterilization are minimized. Contamination is also lower in high temperatures (Roehr 2001; Talukder et al. 2016).

Of the six isolates tested, four isolates namely SL1(1), R5, SL6, and OPT4B had better tolerance to high temperatures with OD values of 2.02, 1.93, 1.89, and 1.58, respectively, at 45 °C, while the other two isolates, namely SL7 and SL3, had lower tolerance to high temperatures with an OD value below 1.00 at 45 °C.

### Xylitol production

**Fig. 6:**
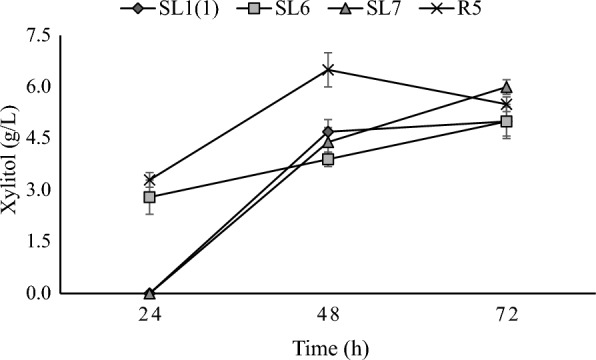
Xylitol production profiles by yeast isolates. Cells were grown in YPX (xylose 50 g/L) media for 72 h at 30 °C and 100 rpm. Two independently replicated experiments generated data points. The variance between repetitions was < 10%

The four isolates tested namely SL1(1), SL6, SL7 and R5 can produce xylitol from xylose substrate in the medium. Xylitol production profiles by yeast isolates are shown in Fig. 6. From the data, the xylose consumed increases with the longer incubation time but increased xylose consumption (Fig. 4) does not always result in higher xylitol production. Xylitol production from yeast isolated from this study compared to other studies is shown in Table 1.

**Table 1:** Xylitol production from yeast isolated from this study compared to other studies

Species/strain	Substrate and fermentation condition	Xylitol production	Reference
*Pichia kudriavzevii* SL1(1)	D-Xylose YPX (50 g/L), 30 °C, 100 rpm, 72 h	5.0 g/L	This research
*Candida xylopsoci* SL6	D-Xylose YPX (50 g/L), 30 °C, 100 rpm, 72 h	5.0 g/L	This research
Isolate SL7	D-Xylose YPX (50 g/L), 30 °C, 100 rpm, 72 h	6.0 g/L	This research
*Pichia kudriavzevii* R5	D-Xylose YPX (50 g/L), 30 °C, 100 rpm, 72 h	5.5 g/L	This research
*Pichia kudriavzevii* UniMAP 3–1	D-Xylose (40 g/L), 30 °C, 150 rpm, 60 h	8.24 g/L	Rahman et al. 2021
*C. tropicalis* E2 *C. tropicalis* S4 *C. tropicalis* FP	Basal medium with xylose (100 g/L) and yeast extract (10 g/L), 30 oC, 120 h	22 g/L 15.5 g/L 4.5 g/L	Martins et al. 2018

The strains isolated in this study produced significantly lower xylitol than the strains of the *Candida tropicalis* species isolated by Martins et al. (2018) and slightly lower than the *Pichia kudriavzevii* strains isolated by Rahman et al. (2021). However, Martins et al. (2018) and Rahman et al. (2021) carried out the incubation time for 120 and 150 days longer than the incubation time used in this study, which was 72 h. The xylitol produced in this study was slightly higher than that of the *Candida tropicalis* species, which was also isolated by Martins et al. (2018). Strains of the same species show different abilities to produce xylitol in the same medium and incubation conditions, so it is necessary always to screen new strains to determine their fermentation potential. The bioconversion of xylose to xylitol in yeast consists of two steps: transfer of xylose into cells followed by reduction from intracellular xylose to xylitol by the enzyme xylose reductase (XR; EC 1.1.1.307) or aldose reductase (EC 1.1.1.21) produced by yeast (Guo et al. 2006).

There are several limitations to using xylose as a bioconversion substrate for xylitol production. The first is that an additional carbon source is required for cell growth. If the carbon source is limited, only part of the xylose is converted to xylitol, and the other part will be used for cell maintenance and growth. The most suitable co-substrate is glucose; however, glucose can inhibit xylose uptake into cells and their metabolism. It is also essential to block the further metabolism of xylitol in the xylitol dehydrogenase reaction so that xylitol is not converted to xylulose. Yeast strains that accumulate high amounts of xylitol express strong XYL1 genes capable of converting xylose to xylitol. Such a strain also has a knockout of the XYL2 gene, which prevents the conversion of xylitol to xylulose (Ruchala and Sibirny 2021).

### Ethanol production

**Fig. 7:**
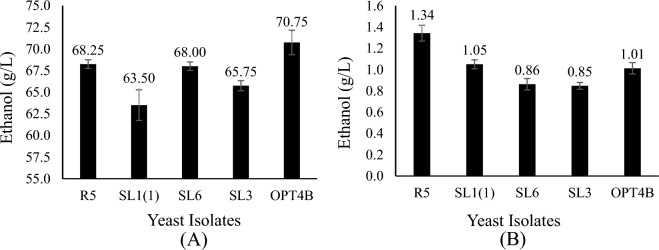
Ethanol production in YPD (glucose 100 g/L) (**A)** and YPX (xylose 50 g/L) (**B)** medium. Cells were cultured in medium at 30 °C, 100 rpm, 72 h. Two independently replicated experiments generated data points. The variance between repetitions was < 10%

*Saccharomyces cerevisiae* is the most commonly used microbe for ethanol production because it can grow in simple carbohydrates like disaccharides. However, it cannot ferment pentoses naturally (Kumar et al. 2009). The five strains isolated in this study could produce ethanol in a medium containing glucose or xylose as the sole carbon sources, as shown in Fig. 7. Ethanol produced in a medium with a carbon source of glucose ranged between 63.50 and 70.75 g/L, while in a medium with a carbon source of xylose, it ranged between 0.85 and 1.34 g/L. Ethanol production from yeast isolated from this study compared to other studies is shown in Table 2.

**Table 2:** Ethanol production from yeast isolated from this study compared to other studies

Species. resource	Substrate and fermentation condition	Ethanol production	References
*Pichia kudriavzevii* SL1(1)	D-Xylose YPX (50 g/L), 30 °C, 100 rpm, 72 h	1.05 g/L	This research
*Pichia kudriavzevii* R5	D-Xylose YPX (50 g/L), 30 °C, 100 rpm, 72 h	1.34 g/L	This research
*Candida xylopsoci* SL6	D-Xylose YPX (50 g/L), 30 °C, 100 rpm, 72 h	0.86 g/L	This research
SL3	D-Xylose YPX (50 g/L), 30 °C, 100 rpm, 72 h	0.85 g/L	This research
OPT4B	D-Xylose YPX (50 g/L), 30 °C, 100 rpm, 72 h	1.01 g/L	This research
*Pichia kudriavzevii* SL1(1)	D-Glucose YPD (100 g/L), 30 °C, 100 rpm, 72 h	63.50 g/L	This research
*Pichia kudriavzevii* R5	D-Glucose YPD (100 g/L), 30 °C, 100 rpm, 72 h	68.25 g/L	This research
*Candida xylopsoci* SL6	D-Glucose YPD (100 g/L), 30 °C, 100 rpm, 72 h	68.00 g/L	This research
SL3	D-Glucose YPD (100 g/L), 30 °C, 100 rpm, 72 h	65.75 g/L	This research
OPT4B	D-Glucose YPD (100 g/L), 30 °C, 100 rpm, 72 h	70.75 g/L	This research
*Pichia kudriavzevii* UniMAP 3–1	D-Glucose (YPD 40 g/L), 30 & 40 °C, 150 rpm, 72 h	12.5 g/L (incubation at 30 °C) 12.5 g/L (incubation at 40 °C)	Rahman et al. 2021
*Pichia kudriavzevii* UniMAP 3–1	D-Xylose (YPX 40 g/L), 30 & 40 °C, 150 rpm	0.5 g/L (incubation at 30 °C) 0.8 g/L (incubation at 30 °C)	Rahman et al. 2021
*Pichia kudriavzevii* IP4	Glucose (YPD 20%), 48 h at 37 and 42 °C	32.05 g/L (incubation at 37 °C) 14.80 g/L (incubation at 42 °C)	Ulya et al. 2020
*Candida tropicalis* E2 *Candida tropicalis* S4 *Candida tropicalis* FP	Basal medium with xylose (100 g/L) and yeast extract (10 g/L), 30 °C, 120 h	3.0 g/L 5.9 g/L 4.6 g/L	Martins et al. 2018
*Pichia kudriavzevii* S26	Glucose 10%, 37 °C, 48 h	40 g/L	Ndubuisi et al. 2018
*Pichia kudriavzevii* NUN-4 *Pichia kudriavzevii* NUN-5 *Pichia kudriavzevii* NUN-6	16% (*w*/*v*) glucose, 40 °C, 135 rpm, 48 h	88.60 g/L 78.52 g/L 77.97 g/L	Pongcharoen et al. 2018
*Candida blankii ATCC 18735* *Candida shehatae NRRL Y-12856* *Candida pseudorhagii sp.* *nov. strain SSA-1542* *Hamamotoa lignophila sp.* *nov. strain SSA-1576* *Meyerozyma guilliermondii* *sp. nov. strain SSA-1522* *Pichia stipitis CBS 5776* *Schizosaccharomyces pombe ATCC 2478* *Sugiyamaella sp.1 nov. strain* *SSA- 159*	D-Xylose (50 g/L), 72 h D-Xylose (50 g/L), 82 h D-Xylose (50 g/L), 48 h D-Xylose (50 g/L), 72 h D-Xylose (50 g/L), 48 h D-Xylose (50 g/L), 65 h D-Xylose (50 g/L), 72 h D-Xylose (50 g/L), 72 h	5.1 g/L 24.0 g/L 14.7 g/L 10.1 g/L 3.8 g/L 22.3 g/L 5.0 g/L 4.6 g/L	Ali et al. 2017 Ali et al. 2017 Ali et al. 2017 Ali et al. 2017 Ali et al. 2017 Ali et al. 2017 Ali et al. 2017 Ali et al. 2017

The strains isolated from this study produced lower ethanol from xylose substrates compared to several strains from *Candida tropicalis* species (Martins et al. (2018), *Candida blankii, Candida shehatae, Candida pseudorhagii sp., Hamamotoa lignophila sp., Meyerozyma guilliermondii, Pichia stipitis, Schizosaccharomyces pombe,* and *Sugiyamaella sp* (Ali et al. 2017). However, isolates from this study produced higher ethanol from xylose substrates compared to several strains from the species *Pichia kudriavzevii* isolated by Rahman et al. (2021).

To know the ability to produce ethanol from other carbon sources, the isolates from this study were also tested for their ability to convert glucose into ethanol. The ethanol produced from glucose substrate by the isolates from this study was higher than that of several strains of the *Pichia kudriavzevii* species isolated by Rahman et al. (2021) and Ulya et al. (2018) but lower than that produced by several strains of the species *Pichia kudriavzevii* isolated by Ndubuisi et al. (2018) and Pongcharoen et al. (2018). The isolates from this study were able to convert hexose and pentose sugars into ethanol so that they have the potential to be utilized in the hydrolysate of lignocellulosic biomass waste, which generally contains various sugar mixtures.

*Pichia kudriavzevii* M12, a commercial yeast strain that metabolizes xylose to produce ethanol, has genes encoding xylose reductase, xylitol dehydrogenase, and xylulokinase, which are responsible for converting xylose to D-xylulose-5-P and then to the pathway of pentose phosphate to generate ethanol (Chan et al. 2012). Some yeast strains cannot produce ethanol because of an imbalance of redox among cofactors NADP and NAD during the production of xylitol and xylulose. The xylose reductase enzyme uses NADPH to convert D-xylose to xylitol, whereas xylitol de hydrogenase primarily uses NAD + , limiting cofactor recycling. Because of this imbalance, xylitol accumulates in the media, and the pathway fails to create ethanol. NADP + recycling can be alleviated by using the respiratory chain. Therefore, cell growth, rather than ethanol generation, may occur depending on the oxygen level (Liang et al. 2013).

### Growth profile of yeast isolates grown in liquid hydrolysate from pretreated oil palm trunk waste

**Fig. 8:**
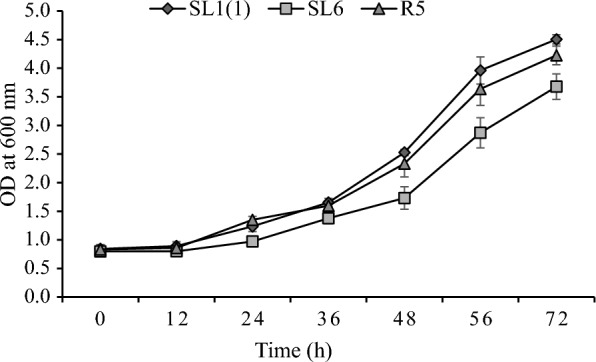
OD profiles of yeast isolates grown in liquid hydrolysate extracted from pretreated oil palm trunk waste. Cells were grown in liquid hydrolysate from pretreated oil palm trunk waste and incubated at 30 °C and 100 rpm. Three independently replicated experiments generated data points. The variance between repetitions was < 10%

Three yeast isolates, namely SL1(1), SL6, and R5 that are of fermenting xylose to produce xylitol and ethanol can grow in liquid hydrolysate produced from pretreated oil palm trunk with the subcritical water method, as shown in Fig. 8. The growth profile of isolate SL1(1), SL6, and R5 reaches growth as measured by cell dry weight (g/L) was 4.34, 4.69, and 5.18, respectively, after 72 h of incubation at 30 °C. The liquid hydrolysate was obtained from pretreated oil palm trunk waste using the subcritical water method and contained xylose and glucose at 4.859 and 4.900 g/L, respectively. The liquid hydrolysate also contains furfural and acetic acid at 0.293 and 2.224 g/L, respectively (data not shown).

Different biomass feedstock and pretreatment methods generate hydrolysates with distinctive toxic compounds, but three major groups of toxic compounds are usually classified in general (Jönsson et al. 2013). These include furan aldehydes, 2-furaldehyde (furfural), and 5-hydroxymethyl-2-furaldehyde (HMF), which are generated by the hydrolysis of pentose and hexose sugars, respectively; aliphatic acids such as particularly acetic acid generated by hemicellulose and lignin deacetylation, formic acid produced by furan decomposition, and levulinic acid obtained by HMF degradation; also included are phenolic substances generated by lignin component degradation (Almeida et al. 2007; Klinke et al. 2004).

From the data in Fig. 8, it can be seen that the growth of yeast isolates in liquid hydrolysate increased significantly after incubation for 48 h, indicating a long lag phase thought to be caused by inhibition of yeast growth due to the presence of these inhibitor compounds. After 48 h, the cells adapted to the conditions in the liquid hydrolysate medium so that a marked increase in growth was seen. The growth of yeast isolates in liquid hydrolysate was more significant than their growth in medium with xylose carbon sources. Although there are inhibitory compounds in the liquid hydrolysate that can inhibit yeast growth, in the liquid hydrolysate, there is a large enough glucose that can be a suitable substrate for yeast cell growth.

Several yeast strains have been studied to tolerate inhibitory compounds produced during the pretreatment of lignocellulosic biomass. Some yeast species that can grow in the hydrolyzate of pretreated lignocellulosic biomass containing inhibitor compounds include *Scheffersomyces (Pichia) stipitis* (Biswas et al. 2013), *Scheffersomyces (Pichia) shehatae* (Senatham et al. 2016), *Candida tropicalis* (Mattam et al. 2016), *Meyerozyma guilliermondii* (Martini 2016), several strains of *Saccharomyces cerevisiae* (Cagnin et al. 2021), several strains of *Saccharomycodes ludwigii, Pichia manshurica,* and *Pichia kudriavzevii* (Pilap et al. 2022).

### Molecular identification of yeast based on the 18S rDNA sequence

**Fig. 9:**
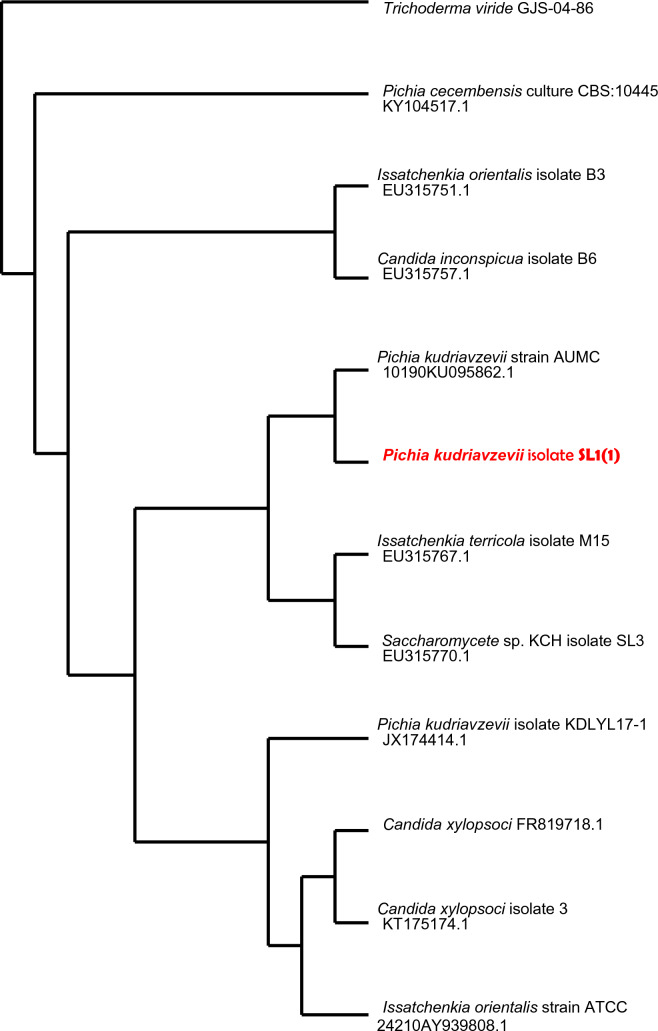
Dendrogram of the phylogenetic relations of the yeast *Pichia kudriavzevii* SL1(1) with other closely related species and strains, obtained from NCBI GenBank. The number written next to the name is the GenBank accession number of the reference strain

**Fig. 10:**
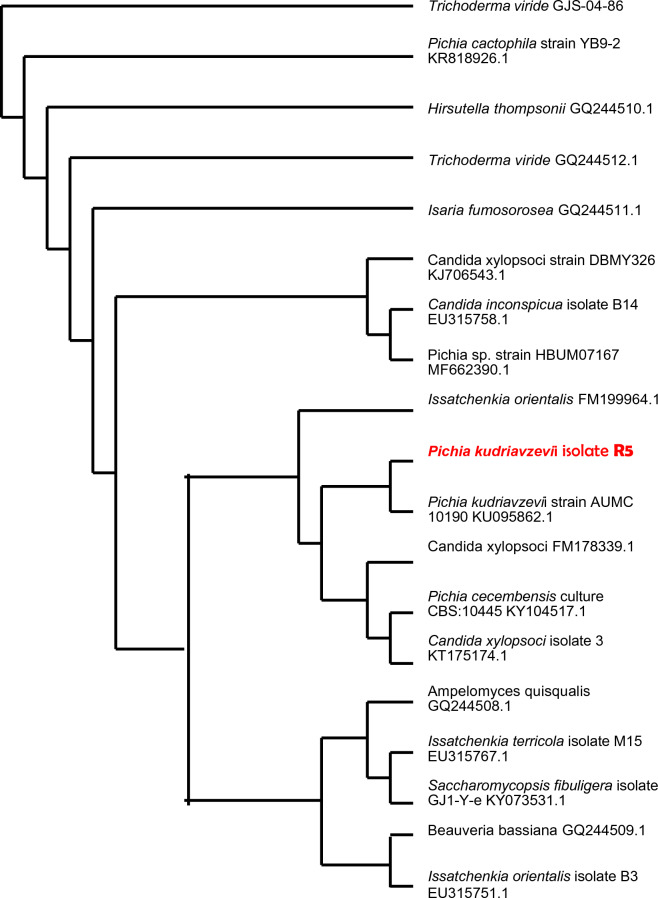
Dendrogram of the phylogenetic relations of the yeast *Pichia kudriavzevii* R5 with other closely related species and strains, obtained from NCBI GenBank. The number written next to the name is the GenBank accession number of the reference strain

**Fig. 11:**
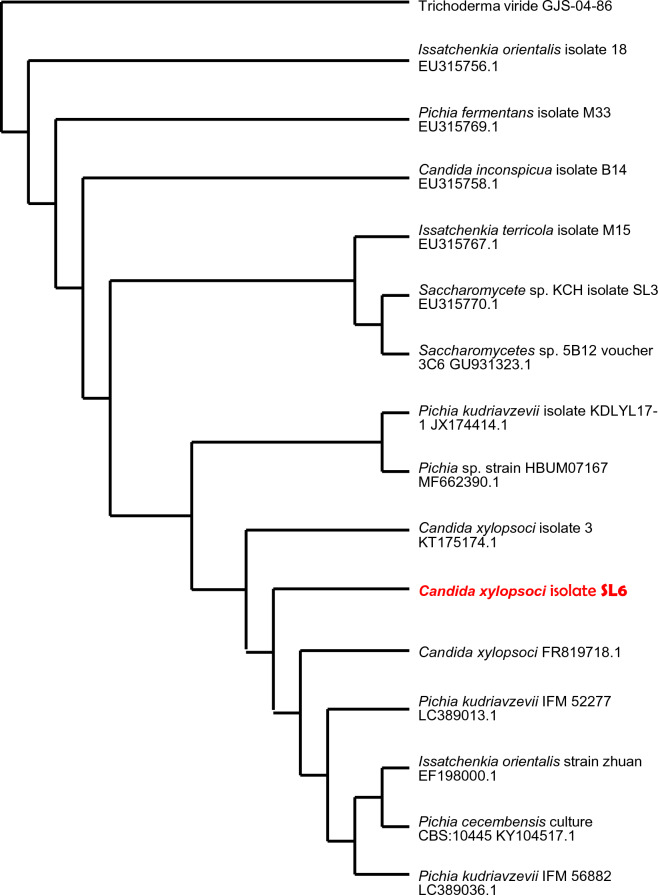
Dendrogram of the phylogenetic relations of the yeast *Candida xylopsoci* SL6 with other closely related species and strains, obtained from NCBI GenBank. The number written next to the name is the GenBank accession number of the reference strain

The preserved 18S ribosomal DNA segment and its diverse flanking area can be utilized to investigate the taxonomic and phylogeny etic connections between closely associated yeast strains and species (Koutinas et al. 2015; Moremi et al. 2020; Wu et al. 2018). The nucleotide sequences of SL1 (1) and R5 were identified as *Pichia kudriavzevii* by blast analysis, whereas the SL6 DNA sequence was similar to *Candida xylopsoci*. Dendrograms of the phylogenetic relationships of yeast isolated in this study with other closely related species and strains obtained from NCBI GenBank based on 18SrDNA sequences are shown in Figs. 9, 10 and 11.

Within the recognized xylose-using microorganisms, yeasts are thought to be among the most effective xylitol or ethanol producers and have received extensive research. The ability to obtain xylitol as a typical metabolic product was proved for different species of yeasts. The fermentation circumstances, including temperature, oxygen, pH, and substrate, which were not investigated in this study, significantly impacted the formation of ethanol and xylitol. Productivity might be enhanced by adjusting the oxygen supply in various fermentation settings (Martins et al. 2018).

## Conclusion

This research found thermotolerant yeasts, namely SL1(1), R5, and SL6, that naturally produce xylitol and ethanol from xylose substrates. These isolates were also able to grow on liquid hydrolyzate from pretreated oil palm trunk waste using the subcritical water method, which contained toxic compounds acetic acid and furan. Based on molecular analysis, yeast isolates capable of producing ethanol and xylitol from pentose carbon sources were identified as *Pichia kudriavzevii* SL1(1), *Pichia kudriavzevii* R5, and *Candida xylopsoci* SL6. These isolates are expected to play a role in producing bioenergy and chemicals from lignocellulosic biomass waste.

## Data Availability

The datasets generated during and/or analyzed during the current study are available from the corresponding author upon reasonable request.

## Abbreviations

YPD: Yeast extract peptone dextrose
YPX: Yeast extract peptone xylose
EC number: Enzyme commission number
NAD: Nicotinamide adenine dinucleotide
NADP: Nicotinamide adenine dinucleotide phosphate
NADPH: Reduced form of NADP
